# Supplementary material for: Investigating the influence of physiologically relevant hydrostatic pressure on CHO cell batch culture
Source: Sci Rep. 2021 Jan 8;11:162. doi: 10.1038/s41598-020-80576-8 (PMC7794228; doi:10.1038/s41598-020-80576-8)
Supplement: Supplementary file 1 — Supplementary Information 1. [file 41598_2020_80576_MOESM1_ESM.zip › S1/IgG/Feb14 0 30 60 90/90mmHg Feb6 2.pdf]

===

Acq. Operator : SYSTEM Seq. Line : 38
Sample Operator : SYSTEM
Acq. Instrument : HPLC-0039 Location : 8
Injection Date : 2/14/2019 4:57:55 PM Inj : 1
Inj Volume : 100.000 µl
Method : C:\Users\Public\Documents\ChemStation\1\Data\TK_CHO_IgG 2019-02-14 14-17-55 \CHO_IgG_LC.M (Sequence Method)
Last changed : 2/8/2019 3:17:23 PM by SYSTEM

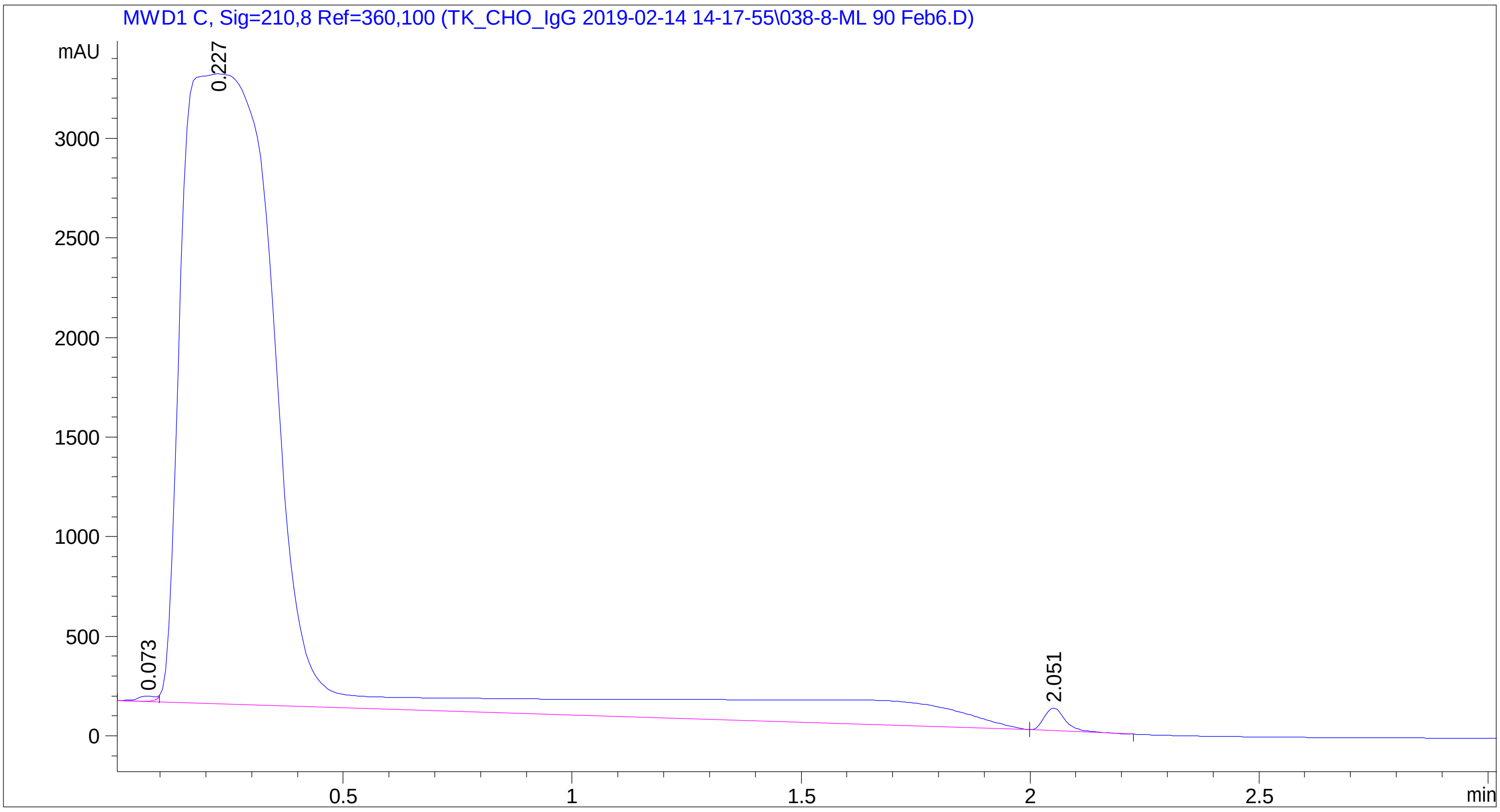

===

Area Percent Report

===

Sorted By : Signal
Multiplier : 1.0000
Dilution : 1.0000
Use Multiplier & Dilution Factor with ISTDs

Signal 1: MWD1 C, Sig=210,8 Ref=360,100

Peak #	RetTime [min]	Type	Width [min]	Area [mAU*s]	Height [mAU]	Area %
1	0.073	BV E	0.0384	63.52738	24.34506	0.1261
2	0.227	VB R	0.1883	4.99409e4	3161.55078	99.1367
3	2.051	BBA	0.0498	371.37689	114.59463	0.7372

Totals : 5.03758e4 3300.49047

===

*** End of Report ***
